# Mental health in hypertension: assessing symptoms of anxiety, depression and stress on anti-hypertensive medication adherence

**DOI:** 10.1186/1752-4458-8-25

**Published:** 2014-06-21

**Authors:** Irene A Kretchy, Frances T Owusu-Daaku, Samuel A Danquah

**Affiliations:** 1 Department of Clinical and Social Pharmacy, Faculty of Pharmacy and Pharmaceutical Sciences, Kwame Nkrumah University of Science and Technology, Kumasi, Ghana; 2 Department of Pharmacy Practice and Clinical Pharmacy, University of Ghana School of Pharmacy, College of Health Sciences, Legon, Ghana; 3 Department of Psychology, University of Ghana, Legon, Ghana

**Keywords:** Hypertension, Negative emotions, Medication non-adherence, Spirituality, Ghana

## Abstract

**Background:**

Patients with chronic conditions like hypertension may experience many negative emotions which increase their risk for the development of mental health disorders particularly anxiety and depression. For Ghanaian patients with hypertension, the interaction between hypertension and symptoms of anxiety, depression and stress remains largely unexplored. To fill this knowledge gap, the study sought to ascertain the prevalence and role of these negative emotions on anti-hypertensive medication adherence while taking into account patients’ belief systems.

**Methods:**

The hospital-based cross-sectional study involving 400 hypertensive patients was conducted in two tertiary hospitals in Ghana. Data were gathered on patient’s socio-demographic characteristics, anxiety, depression and stress symptoms, spiritual beliefs, and medication adherence.

**Results:**

Hypertensive patients experienced symptoms of anxiety (56%), stress (20%) and depression (4%). As a coping mechanism, a significant relation was observed between spiritual beliefs and anxiety (*x*^2^ = 13.352, *p* = 0.010), depression (*x*^2^ = 6.205, *p* = 0.045) and stress (*x*^2^ = 14.833, *p* = 0.001). Stress among patients increased their likelihood of medication non-adherence [odds ratio (OR) = 2.42 (95% CI 1.06 – 5.5), *p* = 0.035].

**Conclusion:**

The study has demonstrated the need for clinicians to pay attention to negative emotions and their role in medication non-adherence. The recommendation is that attention should be directed toward the use of spirituality as a possible mechanism by which negative emotions could be managed among hypertensive patients.

## Background

As disease burdens shift from infectious to non-communicable diseases, hypertension is a principal precursor to cardiovascular diseases and a main cause of death globally [1,2]. About 80% of these deaths were recorded in low- and middle- income countries and projections indicate that the highest non-communicable mortality rates would be recorded in these countries by 2020 [3]. Hypertension affects approximately 25% of urban and 20% of rural Ghanaian populations [4] and 11% - 42% of Africans [2,5-7]. A global hypertension prevalence of 26% is projected to ascend to 29% by the year 2025 [8].

Like patients with other chronic medical conditions, hypertensive patients experience many profound emotions which increase their risk for the development of mental health disorders particularly anxiety and depression [9,10]. Imperative to the management of hypertension is the need for patients to adhere to pharmacological and non-pharmacological therapies and these negative emotions may adversely influence their adherence behaviour [11].

Anxiety and lower adherence rates have been observed for asthma, heart failure, haemodialysis and contraceptive use [12-15], although Kim et al. [16] noticed greater adherence among the majority of their patients with anxiety disorder.

Depression is a burdensome disease of global importance [17] and although prevalent, it is mostly undiagnosed in patients with hypertension [18]. Some relationship has been observed between depression and non-adherence to medical therapy [19,20] and a high number of prescribed medications were listed as one of the contributing factors for the development of depressive symptoms in hypertension [21]. However not all studies successfully showed a relationship between depressive symptoms and adherence [14,22].

The stress of having a chronic medical condition may potentially influence medication adherence behaviour; yet, earlier studies on emotional determinants of adherence have largely focused on depression and anxiety [12,14]. In clinical settings, stress has repeatedly been used as a euphemism for negative emotions, particularly to address undesirable psychiatric diagnostic labelling [23]. Stress negatively influenced medication adherence behaviour in HIV/AIDS [24] and acute coronary syndrome [25]. Empirical evidence showed the importance of stress in the onset and worsening of essential hypertension [26], yet there is a dearth of information associating stress and medication adherence in hypertension management.

Drawing a causal relationship between anxiety, depression and stress, in hypertension and medication adherence may be difficult [19]; but on the other hand, overlooking the association may further decrease attempts to manage the burden of medication non-adherence. For Ghanaian patients with hypertension, this interaction between hypertension and symptoms of anxiety, depression and stress remains largely unexplored and incompletely understood in terms of its prevalence and effect on medication adherence. To fill this knowledge gap, the study sought to ascertain 1) whether hypertensive patients exhibited symptoms of anxiety, depression and stress; 2) whether individuals experiencing anxiety, depression and stress symptoms were more likely to be non-adherent than patients without these symptoms; and 3) whether patients’ belief systems had a relationship with anxiety, depression and stress symptoms.

## Methods

### Study design and setting

A hospital-based cross-sectional study design was used. The study was carried out at the two major teaching hospitals in Ghana; Korle-Bu Teaching Hospital (KBTH), Accra and Komfo Anokye Teaching Hospital (KATH), Kumasi. The description of the study site has previously been reported [27].

### Participants

Two hundred (200) hypertensive outpatients each were recruited from KBTH and KATH. Eligibility to participate in the study was based on the following: A diagnosis of hypertension only or hypertension with other co-morbid conditions, reporting prescription of at least one antihypertensive medication for a minimum of two months and an age of at least eighteen years. The sample did not include pregnant women (because of the possibility of gestational hypertension which may resolve after delivery), newly diagnosed patients as well as the physically and mentally incapacitated [27].

### Measures

After informed written consent, a standardized quantitative assessment tool was used to collect data concurrently from the hypertensive patients attending KBTH and KATH between May and October, 2012. The information gathered covered three areas: i) demographic characteristics; ii) anxiety, depression and stress measures using the Depression Anxiety Stress Scale (DASS) – 21 [28]; iii) medication adherence behaviour using the Morisky Medication Adherence Scale [29]; and iv) the Spiritual Perspective Scale [30]. Participants were asked about their age, sex, place of residence, religious affiliation, marital status, educational level, and duration of hypertensive diagnosis.

The DASS is a 21 item self-report inventory that measures the negative emotional states of depression, anxiety and stress. Each of the three scales comprised seven items with related content. The depression subscale assessed dysphoria, hopelessness, devaluation of life, self-depreciation, and lack of interest/involvement, anhedonia, and inertia. The anxiety subscale measured autonomic arousal, skeletal muscle effects, situational anxiety, and subjective experience of anxious affect. The stress subscale measured relaxation difficulty, nervous arousal, agitation, irritability and impatience. Participants were requested to use a 4-point severity/frequency scale to rate the extent to which they had experienced each negative state over the past week. Reliability for the three scales is 0.71 for depression, 0.79 for anxiety and 0.81 for stress [28]. The DASS anxiety subscale has a correlation coefficient of 0.81 with the Beck Anxiety Inventory whereas the DASS depression subscale had 0.74 with the Beck Depression Inventory [31,32].

The MMAS is an 8-item scale used to measure medication adherence behavior in hypertensive patients and responses are categorized as low adherence (<6), medium adherence (6 - < 8), and high adherence (8). Low and moderate scores were grouped as poor adherence levels [33].

The ten-item SPS measured the belief perceptions of participants relating to spiritually-related interactions. Scores above or below the mean respectively represented high and low spiritual involvement. The SPS has consistently been reliable with Cronbach’s alpha above 0.90 [34].

### Analysis

The data gathered from the study were analyzed with the Statistical Package for Social Sciences (SPSS) version 20. Descriptive statistics were used to represent the characteristics of participants. Anxiety, depression and stress, and medication adherence as well as the level of spirituality were evaluated using chi-square tests and logistic regression models.

### Ethics

Ethical clearance from the institutional ethics committees for KBTH and KATH were obtained before conducting the study. The ethical approval codes are NMIMR-IRB CPN 044/10-11and CHRPE/AP/022/12 respectively.

## Results and discussions

### Sample characteristics

A summary of the sample characteristics (mean and standard deviation) of the hypertensive patients are presented in Table 1. Approximately 63% were women, 33% were 50 to 59 years old, 64% were married, 90% were Christians and 54% had a minimum of a secondary school education. Of these approximately 80% had had hypertension for ≥ 10 years, 42% had co-morbidities and diabetes accounted for 60% of these. Anxiety, depression and stress were not reported in the co-morbid health conditions. This is to be expected as negative emotions are usually not included in the co-morbidities reported by patients [35]; and may partly be due to the lack of adequate medical information being given to the patients. This finding is however interpreted bearing in mind the limitation that those co-morbidities were self-reported and not obtained from the clinicians or from the patients’ health records.

**Table 1:** Characteristics of study sample

**Variable**	**Frequency**	**Percentage**
**Sex**		
Male	149	37.25
Female	251	62.75
**Age**		
< 20	1	0.25
20 - 29	12	3.00
30 - 39	20	5.00
40 - 49	71	17.75
50 - 59	130	
60 - 69	105	26.25
≥ 70	61	15.25
**Marital status**		
Single	39	9.75
Married	254	63.50
Widowed	73	18.25
Divorced/separated	25	6.25
Co-habiting	9	2.25
**Education**		
No formal	48	12.00
Primary	33	8.25
Secondary	217	54.25
Tertiary	102	25.50
**Religious affiliation**		
Christian (spiritual)	18	4.50
Christian (Charismatic/Pentecostal)	166	41.50
Christian (Orthodox)	174	43.50
Muslim	20	5.00
African Traditional Religion	4	1.00
Other	18	4.50
**Number of years of having hypertension**		
≤ 10 years	318	79.50
11 – 20 years	49	12.25
21 – 30 years	20	5.00
31 – 40 years	12	3.00
41 – 50 years	1	0.25
**Level of adherence**		
Low	323	80.75
Moderate	50	12.50
High	27	6.75

### Anxiety and adherence

Anxiety was common among 225 (57%) of the hypertensive patients. This result corroborates the high prevalence of anxiety found among hypertensive patients in varied countries such as South Africa, China and Argentina [10,36,37]; thus showing the presence of anxiety in hypertension in spite of cultural variability. Anxiety in hypertension could result in a higher risk of morbidity and mortality as a result of hastened cardiovascular events [38]. Although it was not measured in the scope of the present study, the anxiety may, in part, be specifically related to the medications or to the chronic health condition [39]. Approximately 93% of participants poorly adhered to their anti-hypertensive medications (Table 1). Although previous studies had correlated anxiety and medication adherence [12,14-16] the present study did not observe any significant association between anxiety and adherence (*x*^2^ = 3.887, *p* = 0.421), [(OR) = 1.6 (0.7 – 3.66), *p* = 0.262]; yet our findings on the prevalence of anxiety calls for clinicians to take critical action in addressing the negative emotional needs of anxious hypertensive patients (Tables 2, 3 and 4).

**Table 2:** Distribution of degree of symptoms of depression, anxiety and stress

**Emotional condition**	**Normal**	**Mild**	**Moderate**	**Severe**	**Extremely severe**
	**N (%)**	**N (%)**	**N (%)**	**N (%)**	**N (%)**
Depression	358 (89.50)	25 (6.25)	12 (3.00)	4 (1.00)	1 (0.25)
Anxiety	129 (32.25)	46 (11.50)	140 (35.00)	44 (11.50)	41 (10.25)
Stress	259 (64.75)	59 (14.75)	52 (13.00)	26 (6.50)	4 (1.00)

A high number of patients experienced moderate to extremely severe symptoms of anxiety (225), followed by stress (82) and depression (17).

**Table 3:** Relationship between negative emotional characteristics and medication non-adherence

**Variable**	**Chi-square**	***p*-value**
Depression	0.004	0.950
Anxiety	3.887	0.421
Stress	5.936	0.037

At p < 0.05, stress and not depression and anxiety significantly related with medication non-adherence.

**Table 4:** A logistic regression model for negative emotional symptoms and medication non-adherence

**Variable**	**OR**	**95%CI**	***p*** **value**
Anxiety symptoms (present: ref absent)	1.6	0.7 – 3.66	0.262
Depressive symptoms (present: ref absent)	0.81	0.1 – 6.29	0.837
Stress symptoms (present: ref absent)	2.42	1.06 – 5.5	0.035

Adjusted for demographic and clinical characteristics.

### Depression and adherence

Moderate to extremely severe levels of depressive symptoms which merit clinical attention were found in 17 (4%) study participants (Table 2). This information is vital because of the increasing impact of depression on the global disease burden [17]. Yet, depression among hypertensive patients is usually not diagnosed [35]. As a consequence such patients may be denied comprehensive clinical care which takes their mental health into consideration. Contrary to other studies which observed participants with depression exhibiting a higher likelihood of medication non-adherence [16,20,40], this association was not observed in the current study (*x*^2^ = 0.004, *p* = 0.950) which draws the same conclusion as studies by Schweitzer et al. [14] and Corvera-Tindel et al. [22]. These studies showed no relationship between the two variables among patients with chronic heart failure. The outcome of a relationship between depression and non-adherence is likely to be inconclusive due to the small number of participants in this study exhibiting symptoms of depression. However, it is essential for clinicians to pay attention to these negative emotional symptoms because overlooking them may further decrease attempts to manage the global burden of chronic diseases.

### Stress and adherence

Studies on emotional determinants of medication adherence have focused primarily on anxiety and depressive symptoms; however significant stressful events have been reported to be responsible for hypertension [41,42]. We reported 82 patients (20%) exhibiting moderate to severe high scores of stress symptoms which may require clinical attention and management (Table 2). Similar to earlier studies on HIV/AIDS [24] and acute coronary syndrome [25], stress was associated with medication non-adherence among the study participants (*x*^2^ = 5.936, *p* = 0.037). The patients who were stressed were more likely to be non-adherent than those who had no or low stress levels [(OR) = 2.42 (1.06 – 5.5), *p* = 0.035]. A probable explanation is that patients showing stress symptoms may be more susceptible to the negative effects of their medications and thus may discontinue taking them. This observation further supports the need for health providers in this area to pay particular attention to medication adherence in patients who are stressed or those who could potentially be affected by stress.

### Relationship between spirituality and anxiety, depression and stress

Spirituality showed a significant association with lower levels of anxiety (*x*^2^ = 13.352, *p* = 0.010), depression (*x*^2^ = 6.205, *p* = 0.045) and stress (*x*^2^ = 14.833, *p* = 0.001) (Table 5). These strong spiritual attributes may have enabled patients cope better with the emotional problems of having a chronic condition like hypertension. Greater spiritual well-being has been found to be associated with fewer symptoms of anxiety, depression and stress [43-46]. Most of these studies related to terminally ill patients yet, the outcome from our study has demonstrated a confirmation of the alleviating role of spirituality in emotional experiences that can be applicable to hypertensive patients and by extension, patients with other chronic conditions.

**Table 5:** Relating spirituality with depression, anxiety and stress symptoms experienced by hypertensive patients

**Variable**	**Depression**		**Anxiety**		**Stress**	
	***X***^**2**^	***p*** **value**	***X***^**2**^	***p*** **value**	***X***^**2**^	***p*** **value**
Spirituality	6.205	0.045	13.352	0.010	14.833	0.001

Some limitations are acknowledged. First, this study was conducted in tertiary hospitals only, thus the views of patients with hypertension in Ghana who seek medical care from other healthcare facilities were not obtained. Second, the use of a subjective measure of medication adherence may give wrong adherence estimations. Future studies could supplement patients’ report on adherence with objective assessments of adherence.

## Conclusions

Patients with hypertension manifested symptoms of anxiety, depression and stress. This implies that the patient’s hypertensive state and perhaps the need for adherence to the anti-hypertensive medications placed psychological demands on their health. Thus, although hypertension could be viewed in itself as a biomedical problem, patients’ experiences with the demands of living as hypertensives resulted in mental health problems. This illustrates the link between biomedical problems and the development of psychological disorders. Further, spirituality helped patients cope with the emotional burden of having hypertension; a chronic disease. Therefore, the need to adopt a multi-faceted perspective towards health delivery in Ghana becomes real in the purview of these findings. The involvement of clinicians, pharmacists, clinical/health psychologists, religious leaders, and nurses thus becomes important in alleviating the problem of non-adherence and invariably improving the quality of life outcomes of hypertensive patients. Attention could be directed toward the use of spirituality as a possible mechanism by which negative emotions are managed among hypertensive patients.

## Competing interests

The authors declare that they have no competing interest.

## Authors’ contributions

IK was involved with research concept, data collection, data analysis, interpretation of results, and writing of manuscript. FO and SD contributed to the research concept and interpretation of results. All authors reviewed and approved the final manuscript.
